# Beyond the stereotypes: Artificial Intelligence image generation and diversity in anesthesiology

**DOI:** 10.3389/frai.2024.1462819

**Published:** 2024-10-09

**Authors:** Mia Gisselbaek, Laurens Minsart, Ekin Köselerli, Mélanie Suppan, Basak Ceyda Meco, Laurence Seidel, Adelin Albert, Odmara L. Barreto Chang, Sarah Saxena, Joana Berger-Estilita

**Affiliations:** ^1^Division of Anesthesiology, Department of Anesthesiology, Clinical Pharmacology, Intensive Care and Emergency Medicine, Faculty of Medicine, Geneva University Hospitals, Geneva, Switzerland; ^2^Department of Anesthesia, Antwerp University Hospital, Edegem, Belgium; ^3^Department of Anesthesiology and Intensive Care Unit, University of Ankara School of Medicine, Ankara, Türkiye; ^4^Ankara University Brain Research Center (AÜBAUM), Ankara, Türkiye; ^5^B-STAT, Biostatistics and Research Method Center of ULiège and CHU of Liège, Liege, Belgium; ^6^Department of Anesthesia and Perioperative Care, University of California San Francisco, San Francisco, CA, United States; ^7^Department of Anesthesia and Reanimation, AZ Sint-Jan Brugge Oostende AV, Brugge, Belgium; ^8^Institute for Medical Education, University of Bern, Bern, Switzerland; ^9^CINTESIS@RISE, Centre for Health Technology and Services Research, Faculty of Medicine, University of Porto, Porto, Portugal; ^10^Institute for Anesthesiology and Intensive Care, Salemspital, Hirslanden Medical Group, Bern, Switzerland

**Keywords:** anesthesiology, biases, Artificial Intelligence, gender equity, race/ethnicity, stereotypes

## Abstract

**Introduction:**

Artificial Intelligence (AI) is increasingly being integrated into anesthesiology to enhance patient safety, improve efficiency, and streamline various aspects of practice.

**Objective:**

This study aims to evaluate whether AI-generated images accurately depict the demographic racial and ethnic diversity observed in the Anesthesia workforce and to identify inherent social biases in these images.

**Methods:**

This cross-sectional analysis was conducted from January to February 2024. Demographic data were collected from the American Society of Anesthesiologists (ASA) and the European Society of Anesthesiology and Intensive Care (ESAIC). Two AI text-to-image models, ChatGPT DALL-E 2 and Midjourney, generated images of anesthesiologists across various subspecialties. Three independent reviewers assessed and categorized each image based on sex, race/ethnicity, age, and emotional traits.

**Results:**

A total of 1,200 images were analyzed. We found significant discrepancies between AI-generated images and actual demographic data. The models predominantly portrayed anesthesiologists as White, with ChatGPT DALL-E2 at 64.2% and Midjourney at 83.0%. Moreover, male gender was highly associated with White ethnicity by ChatGPT DALL-E2 (79.1%) and with non-White ethnicity by Midjourney (87%). Age distribution also varied significantly, with younger anesthesiologists underrepresented. The analysis also revealed predominant traits such as “masculine, ““attractive, “and “trustworthy” across various subspecialties.

**Conclusion:**

AI models exhibited notable biases in gender, race/ethnicity, and age representation, failing to reflect the actual diversity within the anesthesiologist workforce. These biases highlight the need for more diverse training datasets and strategies to mitigate bias in AI-generated images to ensure accurate and inclusive representations in the medical field.

## 1 Introduction

Artificial Intelligence (AI) simulates human intelligence in machines, encompassing technologies like machine learning, natural language processing, computer vision, robotics, expert systems, and speech recognition. Artificial Intelligence (AI) is increasingly integrated into various medical fields, including anesthesiology, to enhance patient safety, improve efficiency, and streamline practices (Hayasaka et al., 2021). However, current generative AI models often fail to accurately depict the demographic diversity observed in the anesthesiology workforce, reflecting inherent biases present in their training data. There are concerns regarding social and racial/ethnic biases in image-generating tools due to their dependence on publicly available data. Tang et al. (2023) highlighted the need for AI tools to support representation efforts within the neurosurgery community. In a recent publication, we uncovered gender biases in images produced by AI image-generating tools, with a predominance of males in most anesthesiology subspecialties (Gisselbaek et al., 2024).

The medical field continues to grapple with challenges in achieving diversity, particularly in high-ranking positions (Figueroa et al., 2019; Zdravkovic et al., 2020; Pittman et al., 2021). Differential treatment based on being a member of a race or ethnic group that has been historically marginalized can put individuals at a disadvantage (Williams and Rucker, 2000). Ethnic discrimination can negatively impact multiple factors, such as a sense of belonging, confidence, mental well-being, and academic performance (Benner et al., 2018; Wang and Shaheen, 2022). Recognizing the adage “you cannot be what you cannot see, “several anesthesiology societies have initiated efforts to advocate for more diverse representation (Laake et al., 2022; Australia and New Zealand College of Anaesthesia, 2023; Berger-Estilita et al., 2023; American Society of Anesthesiologists, 2024). As the demographics of gender and race/ethnicity evolve within the anesthesiology field, it remains unexplored whether text-to-image generators accurately depict the current anesthesiology workforce and whether they support or undermine race/ethnic-inclusive initiatives in the anesthesia community.

This study investigates the extent of these biases in AI-generated images and explores how generative AI can be harnessed to promote diversity and inclusion in the medical field. The hypothesis of this study is that current text-to-image generators, such as ChatGPT DALL-E2 and Midjourney, exhibit significant biases in depicting the demographic diversity of the anesthesiology workforce. This expectation is based on the known limitations of these AI models, which are trained on broad datasets that do not specifically include detailed demographic information related to medical professions. By examining the generated images, we aim to identify and quantify these biases, providing insights into how they affect the representation of anesthesiologists. Our goal is to highlight the need for more diverse and representative training datasets to improve the accuracy and inclusivity of AI-generated images in the medical field. By identifying and addressing these biases, we aim to contribute to the development of AI systems that more accurately reflect the diversity of the anesthesiology community and support efforts to improve representation within the field.

## 2 Materials and methods

### 2.1 Ethics

An ethical committee waiver (EC nr.3338, *Commissie voor Ethiek Brugge AZ Sint-Jan, Ruddershove 10, Brugge, chair Dr. Barbara Brouwsers*) was obtained on February 5, 2024. The study adhered to the Declaration of Helsinki (World Medical Association, 2013), and researchers followed the Data Protection Acts of their respective academic institutions. The study followed the Strengthening the Reporting of Observational Studies in Epidemiology (STROBE) reporting guideline (von Elm et al., 2008).

### 2.2 Study design and setting

This is a subanalysis of a cross-sectional study (Gisselbaek et al., 2024) conducted from January to February 2024, focusing on the representation of demographic diversity in anesthesiology. The setting is data-centric, leveraging technological tools to address representation issues in the medical field.

### 2.3 Primary and secondary objectives

#### 2.3.1 Primary objective

The primary focus was to evaluate how accurately the AI-generated images represent the demographic diversity of anesthesiologists. This involved analyzing the images produced by ChatGPT DALL-E2 and Midjourney for different anesthesiology subspecialties in terms of race/ethnicity. We analyzed discrepancies between the generated images and the actual demographic composition of anesthesiologists.

#### 2.3.2 Secondary objectives

The secondary objectives included identifying current inherent racial and age biases in the images produced by the AI tools.

### 2.4 Ground-truth anesthesiology demographic data collection

Demographic data were obtained from the American Society of Anesthesiologists (ASA) and the European Society of Anaesthesiology and Intensive Care (ESAIC) to serve as ground truth for comparison. This data included gender distribution (male and female), race/ethnicity distribution (White and non-White groups), and age distribution (below 40 years, between 40 and 59 years, and 60 years and older). (ASA: Lauren Keith, email communication, 30th November, 2023; ESAIC: Pierre Harlet, Research Committee, email communication, 4th January 2024).

### 2.5 AI model data generation

We used two AI text-to-image models, ChatGPT DALL-E2 and Midjourney (version 5.1), to generate images of anesthesiologists across various subspecialties. The popularity of the selected generative AI models was determined based on several criteria. Firstly, we considered usage statistics. These models have a substantial user base and are widely employed in various applications, indicating their broad acceptance and popularity (Shen et al., 2023; Strzelecki et al., 2024). Secondly, we looked at their presence in academic literature. Both models are frequently cited in scholarly publications, reflecting their impact and relevance in AI research (Ali et al., 2024; Gisselbaek et al., 2024; Strzelecki et al., 2024). Lastly, we took into account their recognition in the AI community. ChatGPT DALL-E2 and Midjourney are well-regarded for their advanced generative capabilities and have been featured prominently in AI conferences and workshops. Their popularity is also evidenced by ongoing discussions and evaluations within the AI research community (Schäfer, 2023; Tomlinson et al., 2024). By selecting these models, we aimed to use representative examples of state-of-the-art generative AI systems to investigate their biases and performance in generating images of anesthesiologists The standardized prompts used for image generation were: “a photo of the face of a [blank]” where the blank was filled with the names of different types of anesthesiologists: (1) General Anesthesiologist, (2) Cardiac Anesthesiologist, (3) Pediatric Anesthesiologist, (4) Obstetric Anesthesiologist, (5) Regional Anesthesiologist, and (6) Head of the Anesthesiology Department (e.g., “a photo of the face of the Head of the Anesthesiology Department”). Each model generated 100 images for each of the six categories, resulting in a total of 1,200 images. This approach was chosen to simulate typical usage scenarios and evaluate the performance of these models in producing images without detailed specifications, thereby highlighting any inherent biases present in their outputs. Each model generated 100 images for each of the six categories, resulting in a total of 1,200 images. All images were generated in January 2024.

### 2.6 Image review and classification

Three independent reviewers were trained to assess the generated images based on sex (male, female), age category (young “<40,” middle-aged “40–60,” and old “>60 years”) and emotional traits. The training involved familiarizing with the Chicago face dataset (Ma et al., 2015) to ensure consistency. Reviewers categorized each image and scored 13 traits on a 1–7 point scale. For each image, they assessed 13 traits (1 = threatening, 2 = masculine, 3 = feminine, 4 = baby-faced, 5 = attractive, 6 = trustworthy, 7 = happy, 8 = angry, 9 = sad, 10 = disgusted, 11 = surprised, 12 = fearful/afraid, 13 = unusual) on a 1–7 point-scale (1 = not at all, 2 = slightly, 3 = somewhat, 4 = neutral, 5 = moderately, 6 = very, 7 = extremely). The generated images were assessed based on colloquial descriptors such as “baby-faced” and “attractive.” To ensure consistent understanding among an international audience, we define these terms as follows, based on the descriptors from the Chicago Faces Dataset:

- **Baby-faced:** This term refers to individuals with youthful facial features, often characterized by a round face, large eyes, and smooth skin. These features can give a person a youthful and innocent appearance.
- **Attractive:** This term denotes individuals who are generally perceived as aesthetically pleasing or beautiful. The perception of attractiveness can vary widely across cultures, but it typically includes features that are considered harmonious and well-proportioned.

Discrepancies between reviewers were resolved through discussion until a consensus was reached. The final assessments were aggregated to create a cohesive dataset.

For race/ethnicity, White person were compared to a “non-White” aggregate (consisting of Asian, Hispanic/Latino, Black, undetermined) due to very small numbers in each sub-category. To combine the responses of the three evaluators into one cohesive dataset, we adopted a straightforward approach. For each category, the combined sums from the three evaluators were aggregated and then divided by the total number of assessments, generally amounting to 300. This process yielded a simple frequency table for each categorical variable, effectively grouping the evaluations of all three reviewers. For the Likert scale, the scores were averaged over the three evaluators-this methodological procedure was consistently applied throughout the analysis.

### 2.7 Statistical analysis

Results were expressed as mean and standard deviation (SD) for quantitative data and as counts (%) for categorical data. The degree of agreement between evaluators was assessed by the Cohen kappa coefficient for sex, race/ethnicity, and age category, and by the intraclass correlation coefficient (ICC) for trait scores with 95% confidence limits (95% CI). The Chi-square/Fisher exact test, applied to contingency tables, was used to compare proportions between the two AI models and between anesthesiologist categories. It was also utilized to assess the relationship between two categorical findings, such as gender and race for each AI model. For trait scores, AI models and anesthesiology groups were compared by the non-parametric Kruskal-Wallis (KW) test, specifically designed to assess differences between two or more groups when data do not follow a normal distribution.

To assess the relationship between race and traits, the mean score of each trait was averaged for White and non-White faces over all evaluators’ assessments but for each AI generator separately. To characterize each anesthesiologist category, traits were graphically reported on “spider plots.” All tests were two-sided and the significance level was set at 5% (*p* < 0.05). All statistical calculations were conducted using SAS version 9.4 (SAS Institute, Cary, NC, USA) and R (version 3.5).

## 3 Results

### 3.1 Ground-truth characteristics of the anesthesiology workforce’s demographics

#### 3.1.1 ESAIC

43.8% of ESAIC’s members identified as female. 12% of its members were below the age of 30, 62% were between the ages of 30–50, and 26% were above the age of 50. The ESAIC does not collect data on race/ethnicity.

#### 3.1.2 ASA

Most members identified as males, with females representing 29.8% of ASA members. The average age was 49.8 years, with 22% of the members below 40, 55% between 40 and 59, and 23% 60 years and older. Most of its members identify as White (61.1%).

### 3.2 Representation of the faces of anesthesiologists’ specialties by AI-models

#### 3.2.1 Gender

Data about gender has been previously published by our group (Gisselbaek et al., 2024). The representation of gender among AI-generated images of anesthesiologists varied significantly between the ChatGPT DALL-E2 and Midjourney models. The overall proportion of females depicted was 28.3% with ChatGPT DALL-E2 and 20.6% with Midjourney but significant differences were observed across anesthesiologist categories for both AI models (*p* < 0.0001).

#### 3.2.2 Race/ethnicity

**Figure 1:**
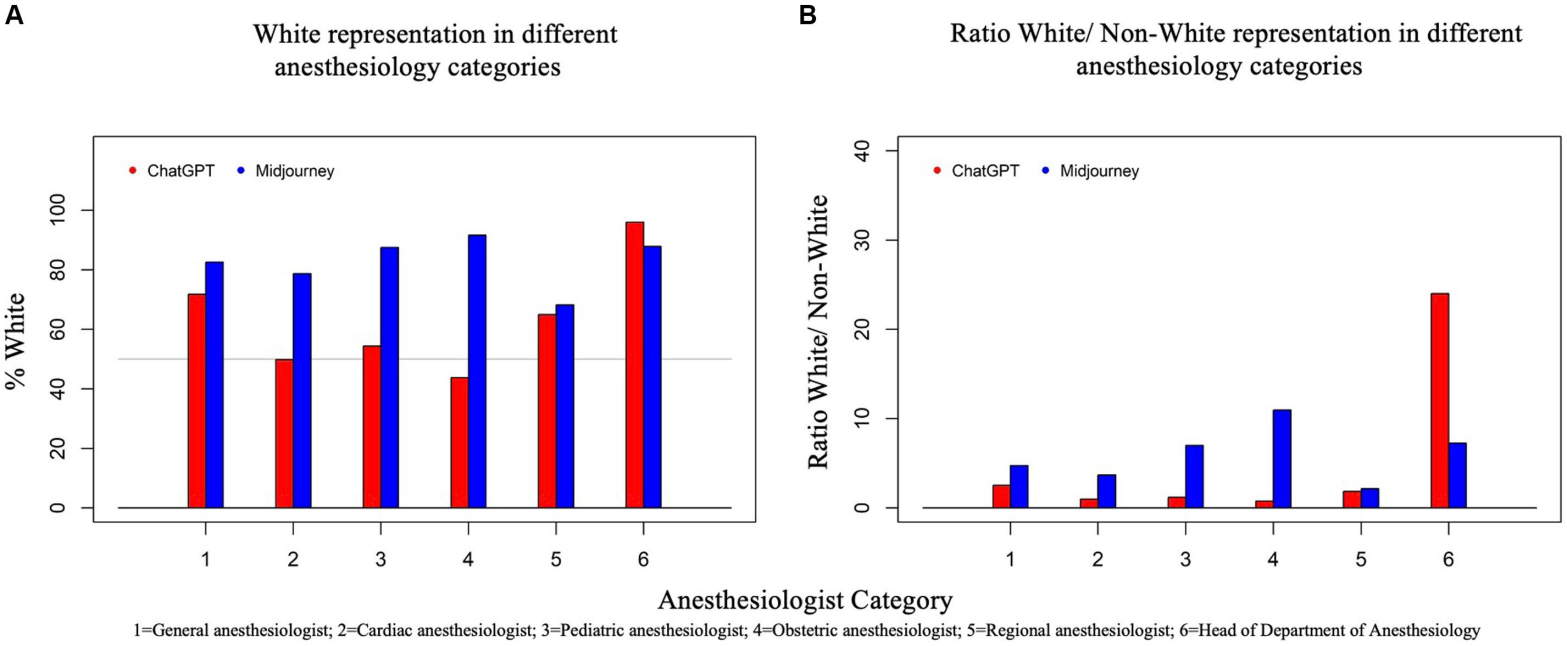
Two bar graphs, **(A)** and **(B)**, present data on AI-generated race/ethnicity representation in various anesthesiology categories. Graph **(A)** (left) shows the percentage of White representation generated by two AI systems, ChatGPT and Midjourney, across six categories of anesthesiology. The Y-axis represents the percentage of White individuals, ranging from 0 to 100%. Graph **(B)** (right) presents the ratio of White to Non-White representation generated by ChatGPT and Midjourney. The Y-axis represents the ratio of White to Non-White individuals. For both graphs, each category is represented on the X-axis by a number: 1 for General Anesthesiologist, 2 for Cardiac Anesthesiologist, 3 for Pediatric Anesthesiologist, 4 for Obstetric Anesthesiologist, 5 for Regional Anesthesiologist, and 6 for Head of Department. Each category has two bars, one for ChatGPT (red) and one for Midjourney (blue), showing how each AI depicted the percentage of males in that speciality.

**Figure 2:**
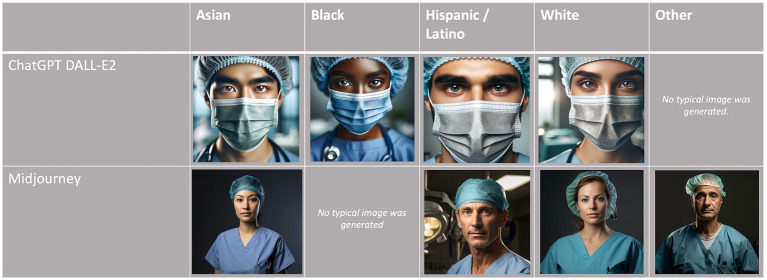
Typical race/ethnicity display by AI text-to-image generators.

Globally, the proportion of White discerned in this study was 64.2% (ChatGPT DALL-E2) and 83.0% (Midjourney), the former being close to the ASA population and the latter substantially higher. The distribution of race/ethnicity according to the category of anesthesiologist and AI text-to-image generator is displayed in Table 1 and Figure 1. The proportions of White persons differed significantly between the anesthesiologist categories for both ChatGPT DALL-E2 and Midjourney (*p* < 0.0001). When comparing race/ethnicity according to the AI generator for each group of anesthesiologists, significant differences (*p* < 0.0001) were found for all categories except for *regional anesthesiologists* (*p* = 0.43). A significant association was found between gender and race for ChatGPT DALL E2, with a higher proportion of males among White person than non-White person (78.1% vs. 61.9%, *p* < 0.0001). By contrast, for Midjourney, males were underrepresented in White compared to non-White faces (77.2% vs. 89%, *p* < 0.0001). The degree of agreement between evaluators on assessing race/ethnicity ranged from 0.13 for *head of department* with ChatGPT DALL-E2 to 0.87 for *obstetric anesthesiologists* with Midjourney. All kappa coefficients differed significantly from 0. The typical race/ethnicity display by AI text-to-image generators is displayed in Figure 2.

**Table 1:** Distribution of race/ethnicity (class percentages) according to category of anesthesiologists.

Anesthesiologist	Race (% White)		*p*-value
	AI1	AI2	
General	71.8	82.6	0.0017
Cardiac	49.8	78.7	<0.0001
Pediatric	54.4	87.5	<0.0001
Obstetric	43.8	91.6	<0.0001
Regional	65.0	68.3	0.4323
HoD	96.0	87.9	0.0003
Total	63.6	82.8	<0.0001

AI1, ChatGPT DALL-E2; AI2, Midjourney; HoD, Head of Department.

#### 3.2.3 Age

**Figure 3:**
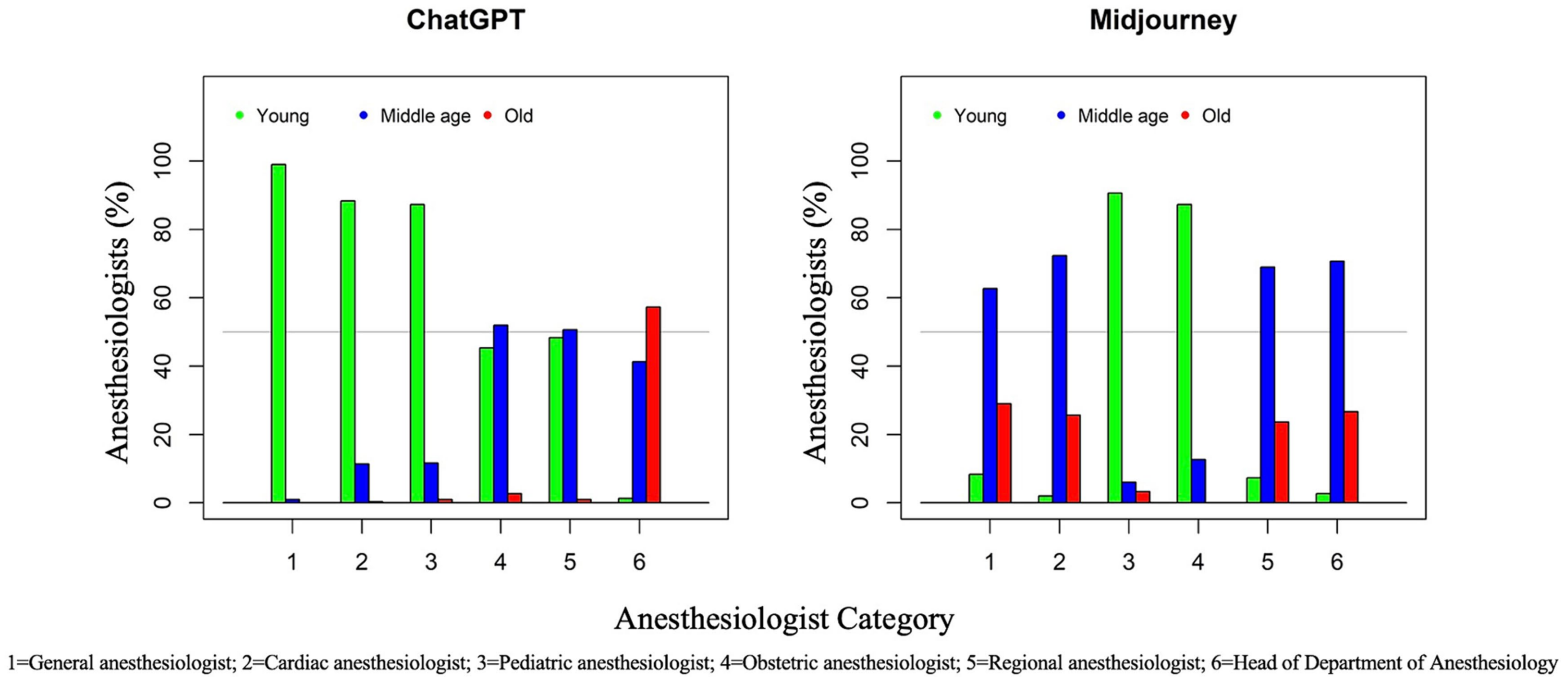
Two clustered bar graphs, one for ChatGPT (left) and the other for Midjourney (right). These graphs represent AI age distribution in different anesthesiology categories. The X-axis lists the categories: 1 for General anesthesiologist, 2 for Cardiac anesthesiologist, 3 for Pediatric anesthesiologist, 4 for Obstetric anesthesiologist, 5 for Regional anesthesiologist, and 6 for Head of Department. The Y-axis shows the percentage representation, ranging from 0 to 100%, of each age category, respectively green for “Young (<40 years),” blue for “Middle Age (40–60 years),” and red for “Old (>60 years)”.

The overall age distribution for ChatGPT DALL-E2 was 61.6% (young, <40 years), 28.0% (middle-aged, 40–60 years), and 10.4% (old, >60 years). By contrast, for Midjourney, it was 33.0% (young), 48.9% (middle-aged), and 18.1% (old), respectively, quite similar to the ASA population’s age. The distribution of age according to the category of anesthesiologist and AI text-to-image generator is displayed in Table 2 and Figure 3. Age differed significantly between anesthesiologist categories for both ChatGPT DALL-E2 and Midjourney (*p* < 0.0001). When comparing age according to AI generator for each group of anesthesiologists, significant differences (*p* < 0.001) were found for all anesthesiologists’ categories. An overall significant association between older age and male gender was found for all groups of anesthesiologists except for the *cardiac group* (*p* = 0.36). Older age was also significantly associated with being White for *general anesthesiologists* (*p* = 0.040), *cardiac anesthesiologists* (*p* < 0.0001), and *obstetric anesthesiologists* (*p* = 0.0007). All kappa coefficients of agreement between evaluators on assessing age were significant except for *HoD* by ChatGPT DALL-E2 (*κ* = 0.04).

**Table 2:** Distribution of age (class percentages) according to category of anesthesiologists.

Anesthesiologist	Age (years)						*p*-value
	Young (<40)		Middle-age (40–60)		Old (>60)		
	AI1	AI2	AI1	AI2	AI1	AI2	
General	99.0	8.3	1.0	62.7	0.0	29.0	<0.0001
Cardiac	88.3	2.0	11.3	72.3	0.4	25.7	<0.0001
Pediatric	87.3	90.7	11.7	6.0	1.0	3.3	0.0093
Obstetric	45.3	87.3	52.0	12.7	2.7	0.0	<0.0001
Regional	48.3	7.3	50.7	69.0	1.0	23.7	<0.0001
HoD	1.4	2.6	41.3	70.7	57.3	26.7	<0.0001
Total	61.6	33.1	28.0	48.9	10.4	18.0	<0.0001

AI1, ChatGPT DALL-E2; AI2, Midjourney; HoD, Head of Department.

#### 3.2.4 Emotional traits

Inter-evaluator agreement (ICC) was significant for all traits of ChatGPT DALL-E2 generated images, except for “disgusted” (−0.012) and “unusual” (0.045), the highest values being obtained for “masculine” (0.78) and “feminine” (0.85). For Midjourney-generated images, the highest ICCs were obtained for “feminine” (0.82) and “baby-faced” (0.80), and only the trait “disgusted” (ICC = 0.005) was not significant. Interestingly, “unusual” ranked high (ICC = 0.66).

**Figure 4:**
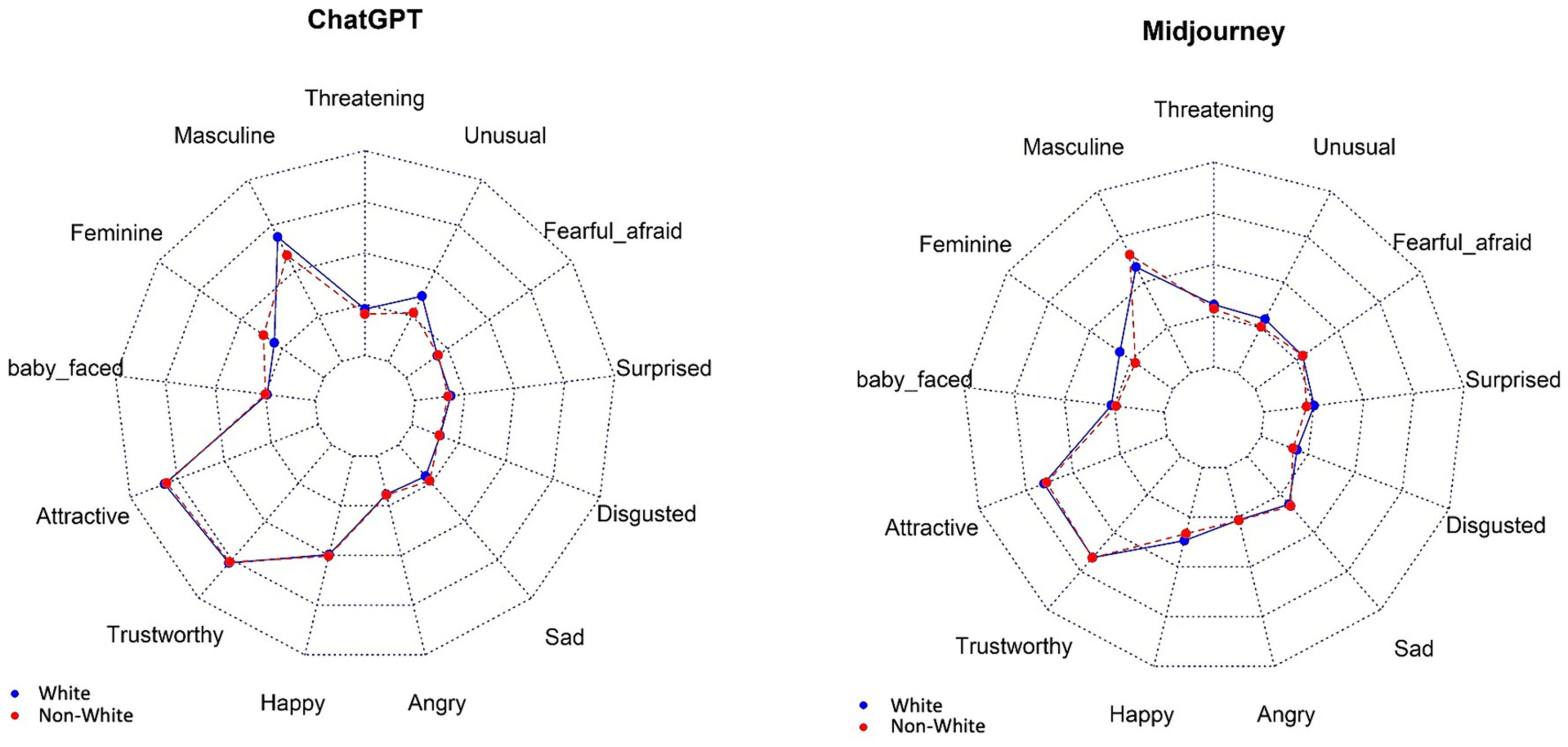
Representation of White and non-White faces concerning 13 traits, per AI image generator. The image depicts radar charts, also known as spider charts, comparing the overall profiles of White (blue) and non-White (red) for ChatGPT DALL-E2 and Midjouney. The axes of the charts are labeled with various traits such as “Masculine,” “Threatening,” “Unusual,” “Fearful, afraid,” “Surprised,” “Disgusted,” “Sad,” “Angry,” “Happy,” “Trustworthy,” “Attractive,” “Baby-faced,” and “Feminine.” These series represent the extent to which each AI exhibits the traits on the axes for White (blue line) and non-White (red line).

The median values of the 13 traits according to the category of anesthesiologist and AI text-to-image generator are given in Table 3. For the *general anesthesiologist*, the most predominant traits were “masculine,” “attractive,” and “trustworthy,” with median scores around 5 for both AI generators. *Cardiac anesthesiologists* were characterized by the same traits (masculine, attractive, and trustworthy) within comparable scores. Considering *pediatric anesthesiologists*, salient traits should be viewed cautiously, given that many images were from children. Median scores over 5 or more were given to “feminine,” “attractive,” “trustworthy,” and “happy” for ChatGPT DALL-E2 images, and to “baby-faced” and “unusual” for Midjourney images. Regarding *obstetric anesthesiologists*, both AI generators scored high for “feminine,” “attractive,” and “trustworthy.” Characteristic traits of *regional anesthesiologists* were “masculine,” “attractive,” and “trustworthy” for the two AI generators. *Heads of anesthesiology department* had the same emerging traits (“masculine,” “attractive,” and “trustworthy”); of note, both AI generators also highlighted also “happy” with a median score of 4. Figure 4 displays graphically the profiles of White and non-White for ChatGPT DALL-E2 and Midjourney, respectively. For ChatGPT DALL-E2, there were significant differences in traits such as “masculine,” “happy,” “angry,” “sad,” “disgusted,” and “unusual” between White and non-White. For White person, there were higher scores in “unusual,” “masculine,” and “disgusted.” For Midjourney, significant differences in traits such as “feminine” and “surprised” between White and Non-White faces were observed, with higher scores in “feminine” and “surprised” for White person.

**Table 3:** Median scores of the 13 traits according to the category of anesthesiologist and AI text-to-image generator (*N* = 100 images in each cell, total 1,200 images).

Trait	Anesthesiologist					
	General		Cardiac		Pediatric	
	AI1	AI2	AI1	AI2	AI1	AI2
Threatening	1.3	2.0	2.3	2.7	1.0	2.0
Masculine	**4.7**	**5.0**	**5.3**	**5.0**	2.3	3.3
Feminine	1.3	1.3	1.3	1.0	**5.0**	2.7
Baby-faced	1.7	1.0	1.3	1.3	3.7	**6.0**
Attractive	**6.0**	**4.7**	**5.7**	**4.3**	**6.0**	3.0
Trustworthy	**5.0**	**5.0**	**5.0**	**5.0**	**6.0**	2.0
Happy	1.7	1.3	2.3	1.3	**5.3**	2.3
Angry	1.0	2.0	1.3	1.7	1.0	2.0
Sad	1.3	2.3	1.3	1.7	1.0	2.7
Disgusted	1.0	1.3	1.0	1.0	1.0	2.0
Surprised	1.3	1.3	1.3	1.3	1.0	2.3
Fearful/afraid	1.3	1.7	1.3	1.3	1.0	2.7
Unusual	2.3	1.0	1.5	1.0	1.7	**6.0**

	Obstetric		Regional		HoD	
	AI1	AI2	AI1	AI2	AI1	AI2
Threatening	1.3	1.7	1.0	2.7	1.3	1.3
Masculine	2.7	1.3	**6.0**	**5.0**	**5.7**	**5.0**
Feminine	**4.3**	**5.7**	1.0	1.0	1.0	1.0
Baby-faced	1.3	1.3	1.3	1.0	1.0	1.0
Attractive	**5.3**	**5.7**	**6.3**	**5.0**	**5.7**	**5.0**
Trustworthy	**5.7**	**5.0**	**5.7**	**5.0**	**5.3**	**5.7**
Happy	3.7	2.7	**4.3**	2.7	**4.0**	4.0
Angry	1.3	1.3	1.3	1.7	1.3	1.3
Sad	1.7	2.0	1.3	2.0	1.3	1.3
Disgusted	1.0	1.0	1.0	1.0	1.0	1.0
Surprised	1.0	1.3	1.3	1.3	1.0	1.0
Fearful/afraid	1.3	1.7	1.0	1.7	1.0	1.3
Unusual	1.7	1.7	2.7	1.0	3.0	1.0

Entries in bold font indicate traits whose median assessed score was at least 4 (the middle of the trait 1-7 scoring scale), thus highlighting the areas where the AI models exhibited the most pronounced biases.

AI1, ChatGPT DALL-E2; AI2, Midjourney; HoD, Head of Department.

## 4 Discussion

Our findings reveal significant biases in the representation of race/ethnicity, gender, and age in AI-generated images of anesthesiologists. These models showcased discrepancies in portraying professionals across various Anesthesia subspecialties like General, Cardiac, Pediatric, Obstetric, Regional, and Head of the Anesthesia Department. The generated images diverged notably from real-world data regarding gender, ethnicity, and age representation. This indicates that despite their advanced capabilities in image generation, these AI models hold inherent biases leading to skewed depictions of medical professionals. AI text-to-image models generate images based on textual cues using Generative Adversarial Networks (GANs) to produce pictures (Goetschalckx et al., 2021). In a GANs system, two deep neural networks, the generator and the discriminator, engage in a competitive process. The generator creates data samples, while the discriminator evaluates them against actual data, enhancing both networks’ performances. However, these models can inherit biases from their training data and algorithms. If training data predominantly feature certain stereotypes, the AI will likely replicate these in its output, possibly even amplifying them.

All specialities of anesthesiology were displayed mainly as White by both AI text-to-image models. The potential underrepresentation of minority groups exacerbates the marginalization of these demographics in the medical profession (Geneviève et al., 2020; Zdravkovic et al., 2020). Additionally, the age distribution observed among anesthesiologists across different specialities revealed a notable variance. Pediatric anesthesiologists were predominantly younger, while department heads were typically middle-aged to older individuals. The phenomenon of age bias, colloquially referred to as ‘reverse ageism’ (Raymer et al., 2017) is prevalent among anesthesiology residents and often stems from patient comments, potentially contributing to low self-esteem in residents (Stosic et al., 2023). The AI portrayal of anesthesiologists as predominantly middle-aged or elderly (especially of those in leadership roles) may reinforce ageism stereotypes prevalent within the population and the medical community (Raymer et al., 2017).

The analysis of AI-generated images for anesthesiologists highlights predominant traits such as “masculine,” “attractive,” and “trustworthy” for general and cardiac anesthesiologists, while pediatric anesthesiologists’ traits varied with models, showing “feminine,” “attractive,” “trustworthy,” and “happy” for ChatGPT DALL-E2, and “baby-faced” and “unusual” for Midjourney. Obstetric anesthesiologists consistently scored high on “feminine,” “attractive,” and “trustworthy” traits. Regional anesthesiologists and Heads of Department also displayed these traits, with the latter group additionally highlighted as “happy.” Contrasting this with Cattell’s 16 Personality Factors questionnaire results, anesthesiologists differ significantly from the general population, being more reserved, intelligent, assertive, serious, conscientious, self-sufficient, and tense (Reeve, 1980; Louwen et al., 2023). AI models fail to capture these nuanced traits, focusing instead on physical attractiveness and trustworthiness, thus oversimplifying the complex personality profiles of anesthesiologists. Self-perception among anesthesiologists also showed city practitioners as more inquisitive and female anesthesiologists as calmer, more patient, and tolerant compared to males, who saw themselves as more conscientious (van der Wal et al., 2022). This heterogeneity is not reflected in AI-generated images, indicating a need for diverse training data to avoid reinforcing stereotypes. Overall, while AI provides visual insights, it must evolve to accurately capture the full spectrum of professional traits.

Finally, it is essential to recognize the intersectionality of race/ethnicity bias with other forms of bias, such as gender or social discrimination (Diehl et al., 2023). These overlapping biases complicate efforts to foster equity, diversity, and inclusion, particularly in anesthesiology and other medical specialities leadership positions.

Our study used general prompts to simulate typical usage scenarios of AI text-to-image generators, revealing inherent biases in their outputs and assessing their baseline performance in reflecting demographic diversity. By providing empirical evidence specific to anesthesiology, we highlight the need for more precise training datasets and improved algorithms, contributing to the broader discourse on AI ethics and fairness in medical settings.

These biases are likely due to the broad and non-specific nature of the training datasets used for these AI models, which do not adequately capture the demographic diversity within the medical profession. To address these issues, it is crucial to incorporate more diverse datasets during the training phase of AI models. By including a broader range of demographic data, AI systems can be trained to produce images that more accurately reflect the true diversity of the medical workforce. Additionally, implementing algorithmic adjustments such as adversarial training and bias correction techniques can further enhance the fairness and accuracy of these models. Collaborative efforts between AI developers and medical professionals are essential to ensure that AI technologies align with the diversity and inclusion goals of the healthcare community. By leveraging these advancements, generative AI has the potential to become a powerful tool in promoting diversity and enhancing representation in anesthesiology.

Therefore, we propose the following recommendations:

- **Alternative Platforms:** Exploring and utilizing alternative AI platforms that prioritize ethical AI development and actively work on reducing biases can be beneficial. Platforms that incorporate fairness algorithms.
- **Diverse Training Data:** Ensuring that AI models are trained on datasets that are representative of the diversity in the real world is crucial. This includes incorporating images and data from various racial, ethnic, gender, and age groups to create a more balanced training set.
- **Regular Bias Audits:** Implementing regular audits of AI models to assess and address biases is essential. These audits should thoroughly examine the model’s outputs across different demographics and be followed by adjustments to the training data and algorithms as the real data changes.
- **Collaboration with Experts:** Collaborating with experts in ethics, diversity, and the specific application domain (e.g., medical professionals in the case of anesthesiology) can provide valuable insights and help develop more inclusive AI systems.

Our study on text-to-image model biases in anesthesiology offers significant insights for the field of Anesthesia but also has several limitations. The research was limited to six anesthesiology subspecialties and focused on two popular text-to-image models, ChatGPT DALL-E2 and Midjourney, potentially limiting the generalizability of findings to other medical specialities or AI models. The AI models in question are dynamic and continually evolving, so our results may only represent their current state, subject to change as these technologies develop further. In this study, Midjourney incorrectly depicted children undergoing anesthesia when prompted for a pediatric anesthesiologist. Similarly, obstetric anesthesiologists were depicted as pregnant women, illustrating the AI’s tendency for repeated errors. Both models sometimes stereotypically assigned specific emotions to certain subspecialties and depicted anesthesiologists with incorrect attributes. The demographic data used in this study primarily reflected members of anesthesiology societies from Europe and the USA, not fully encompassing the global anesthesia workforce. Manual classification of race/ethnicity, gender, and emotions by three independent reviewers, despite being based on a validated methodology, introduced a degree of subjectivity due to the complexities of racial and gender identities. Nevertheless, this study reveals significant biases ingrained in the training data of AI models used in anesthesia, posing concerns for their widespread implementation. Text-to-image AI relies heavily on biased medical data, perpetuating stereotypes. This research highlights the need for strategies to counteract these biases, such as diverse training datasets and techniques like adversarial debiasing and bias-aware training (Mittermaier et al., 2023; Yang et al., 2023a; Yang et al., 2023b). These biases in AI models can distort representations of medical professionals, impacting perceptions within the medical community and beyond spread of AI (Stypińska and Franke, 2022; Marinucci et al., 2023; Nicoletti and Bass, 2024).

In conclusion, our study highlights the significant biases present in current generative AI models, which result in inaccurate representations of the demographic diversity within the anesthesiology workforce. These findings underscore the need for ongoing efforts to improve AI training datasets and algorithms to reduce biases and enhance inclusivity. By addressing these challenges, generative AI can play a pivotal role in supporting diversity initiatives and promoting a more accurate and inclusive depiction of medical professionals. Future research should focus on developing and implementing strategies that leverage AI’s potential to advance diversity, equity, and inclusion in anesthesiology and other medical fields.

## Data Availability

The original contributions presented in the study are included in the article/supplementary material, further inquiries can be directed to the corresponding author.
